# Missing covariate data within cancer prognostic studies: a review of current reporting and proposed guidelines

**DOI:** 10.1038/sj.bjc.6601907

**Published:** 2004-06-08

**Authors:** A Burton, D G Altman

**Affiliations:** 1 Cancer Research UK Clinical Trials Unit, University of Birmingham, Birmingham, B15 2TT, UK; 2 Cancer Research UK/NHS Centre for Statistics in Medicine, Oxford, OX3 7LF, UK

**Keywords:** missing covariate data, reporting, prognostic models, guidelines, survival

## Abstract

Prognostic models play a crucial role in the clinical decision-making process. Unfortunately, missing covariate data impede the construction of valid and reliable models, potentially introducing bias, if handled inappropriately. The extent of missing covariate data within reported cancer prognostic studies, the current handling and the quality of reporting this missing covariate data are unknown. Therefore, a review was conducted of 100 articles reporting multivariate survival analyses to assess potential prognostic factors, published within seven cancer journals in 2002. Missing covariate data is a common occurrence in studies performing multivariate survival analyses, being apparent in 81 of the 100 articles reviewed. The percentage of eligible cases with complete data was obtainable in 39 articles, and was <90% in 17 of these articles. The methods used to handle incomplete covariates were obtainable in 32 of the 81 articles with known missing data and the most commonly reported approaches were complete case and available case analysis. This review has highlighted deficiencies in the reporting of missing covariate data. Guidelines for presenting prognostic studies with missing covariate data are proposed, which if followed should clarify and standardise the reporting in future articles.

Prognostic models formalise the multivariate relationships between multiple patient characteristics and outcome and can be useful tools to aid clinical decision-making. The construction of a prognostic model ideally requires a large database with complete information on all potential prognostic factors. However, often some cases have missing covariate data, which may introduce bias and lead to misleading conclusions if handled inappropriately.

There are many strategies available for handling missing data (Little and Rubin, 1987; Schafer and Graham, 2002). These include the simple deletion approaches of complete case analysis, where only the cases with complete data for all collected variables are analysed, available case analysis, where the cases with complete data for the variables in the fitted model are analysed utilising the largest possible data set, and variable omission (Vach, 1997), where the incomplete variable is excluded from the model. Other techniques, utilising all cases, include analysing the missing data as a separate category (Greenland and Finkle, 1995), single imputation (Little and Rubin, 1987), in which a single value is substituted for each missing value, and multiple imputation (Rubin, 1987; Schafer, 1997), where more than one independently completed data sets are obtained. Each strategy for handling missing data has an underlying assumption regarding the missing data mechanism (Little and Rubin, 1987), that is, the reasons for the occurrence of the missing covariate data, which if not satisfied could result in biased parameter estimates. For example, the commonly used complete case analysis assumes that the missingness in the covariates is not associated with the outcome (Vach and Blettner, 1998). Most single imputation and multiple imputation approaches assume that the missingness is related to the observed data but does not depend on the unobserved value itself.

A review of cancer studies was undertaken to assess the quality of reporting missing covariate data when constructing prognostic models, to establish the extent of missing covariate data, and to review the current handling of this potentially serious problem. Guidelines for reporting prognostic studies with missing covariate data are proposed.

## METHODS

A sample of 100 articles was obtained using fairly broad inclusion criteria. Articles were included if they were published in 2002 within the seven non-review clinical cancer journals with the highest impact factors; for each journal, searching ceased if 20 articles were identified. A multivariate survival analysis must have been performed to establish the factors affecting prognosis, with at least four covariates considered for potential inclusion. Prognosis could be measured in terms of overall survival, disease-specific survival or disease-free survival. There were no restrictions placed on the sample size, the type of cancer studied or the type of study, allowing both prognostic modelling and prognostic factor studies to be included.

The seven journals were hand searched to identify articles that met the inclusion criteria. The journals, with the associated number of articles obtained from each in brackets, were the Journal of the National Cancer Institute (nine articles), Journal of Clinical Oncology (20 articles), International Journal of Cancer (16 articles), British Journal of Cancer (12 articles), Cancer (20 articles), European Journal of Cancer (11 articles) and Annals of Oncology (12 articles).

A data extraction form was designed to collect the required information and one reviewer extracted all the data. For articles reporting more than one multivariate survival analysis, preference was placed on the results for overall survival, using the whole sample and not a subgroup and on the results for the construction sample and not the validation set.

## RESULTS

The 100 articles described studies in a range of cancer sites, the most common sites being breast cancer (25 articles) and cancers of the digestive organs (22 articles). The articles reported the results from cohort studies (76 articles), randomised trials (11 articles), selection of cases from one or more randomised trials (10 articles), case-controlled studies (two articles) and a case–cohort study (one article). Results were extracted from analyses of overall survival (81 articles), disease-specific survival (11 articles) and disease-free survival (eight articles).

Table 1

**Table 1:** Summary of reporting key aspects of multivariate modelling

**Feature reported**	**Number of articles (*n*=100)**
Total number of eligible cases	87
Total number of eligible events	48
Choice of candidate variables	69
Number of candidate variables	79
Cox proportional hazards model used	98
How covariates handled in analysis	29
Verifying model assumptions	23
Modelling selection strategy	53

provides a summary of the key data and multivariate modelling issues. The total number of eligible cases, satisfying the individual study's inclusion criteria, was reported in 87 articles, and ranged from 29 to 20 561 patients. The amount of censoring ranged from 8 to 93% for the 48 articles where this could be ascertained.

All articles employed Cox proportional hazards modelling with the exception of two: one performed a regression tree analysis (Ciampi *et al*, 1995) and the other used an accelerated failure time model (Harrell, 2001). The choice of candidate variables for potential inclusion in the multivariate survival analysis was discussed in 69 articles. In five of these articles the variables with a large amount of missingness, that is, >25% of values, were excluded from potential inclusion in the model. The number of candidate variables investigated was obtained from 79 articles and ranged from two to 23 variables with a median of six variables.

### Missing covariate data

**Figure 1:**
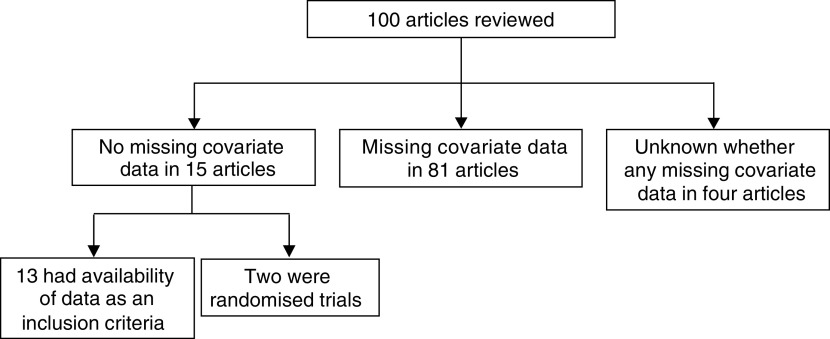
Flow diagram showing the presence or absence of missing covariate data.

Overall, the presence or absence of missing covariate data could be established in 96 articles (Figure 1

). There was insufficient information in the remaining four articles, since there was no patient characteristic table (one article), not all variables were reported (one article) or only summary measures were provided (two articles). Complete data were reported in 15 articles; two articles reported the results from randomised trials and the remaining 13 were retrospective selections of patients with tumour specimen available or specific tests undertaken. Thus, missing data was an exclusion criterion in these 13 studies, but the actual amount of missingness was not specified. In the remaining 81 articles, there was evidence of some missing covariate data. Additionally, in 27 of these 81 articles, there was an inclusion criterion of availability of tumour specimens or data, with 11 articles not specifying the number of cases this criterion excluded.

Table 2

**Table 2:** Summary of reporting missing covariate data

**Feature reported**	**Number of articles (*n*=100)**
Proportion of complete cases	39
Number of incomplete variables	68
Maximum missingness in any variable	70
Amounts of missingness for all variables	52
Methods for handling missingness	32
Reasons for missingness	21
Comparison between complete cases and those with incomplete data	10
Specific missing data section	1

provides a summary of the reporting of missing covariate data within these 100 reviewed articles. The percentage of complete cases out of the total number eligible was extracted from 39 articles as a means to quantify the amount of cases with any missing covariate data. In 52 articles the amounts of missingness, if any, were specified for all the reported variables; 15 with no missing data reported and 37 with some missingness. The extent of missing data was established solely from the text in one article and from tables provided in the remaining 51 articles. The information reported within the tables was either the total number of cases with data for each covariate (eight articles), the observed number of cases within each category (31 articles) or a separate unknown category (12 articles). In one article, the total number of cases with data was supplemented by the percentage of missing data. For 48 articles the full extent of the missingness could not be determined, as there was either no patient characteristics table (eight articles) or insufficient information in the tables (40 articles).

### Quantification of completeness of covariate data

The percentage of eligible cases with complete data was less than 90% in 17 of the 39 articles, where this was determined. The minimum percentage of complete cases was 41%. The number of incomplete variables ranged from none in 15 articles to 30 incomplete covariates in one article, with a median of three incomplete variables reported per article. Also, the variable with the maximum amount of missingness had more than 10% of its values missing in over half of the 70 articles, where this was ascertained (36 articles). The maximum amount of missingness in any one variable was 72%.

### Approaches for handling missing covariate data

In 32 (38%) of the 81 articles with known missing data, at least one method for handling covariates with missing data was mentioned (Table 2), although five of these 32 articles only reported the handling of some, but not all, of the incomplete covariates. The most commonly reported approaches were complete case analysis (12 articles) and available case analysis (12 articles). In addition, six articles omitted between one and four variables due to missing data and four articles included cases with missing values as a separate category within the modelling procedure. In three of the reviewed articles the authors applied an informal single imputation approach, substituting the missing values with values from surrogate variables (two articles) or with the median values calculated from the non-missing data for the covariates in the final model (one article). Multiple imputation was reported in just one article.

The number of cases analysed and associated number of events were given in four of the 32 articles where the approach used to handle the missing data could be determined, and in a further 11 articles where there were no missing data. However, the number of events per variable considered was only sufficient to provide reliable and unbiased results, that is, greater than 10 events per candidate variable (Peduzzi *et al*, 1995), in five of these 15 articles.

### Exploration of the missing data mechanism

The possible reasons for any missingness were discussed in 21 articles (Table 2), the main reason being the lack of availability of tissue samples (17 articles). Other reasons were that the covariate was not collected by design for all patients in the study (two articles) or the inability to determine a response due to contraindications to the required procedure (two articles).

The existence of any differences between the complete and incomplete cases was discussed in 10 articles (Table 2); six of these articles considered differences in patient and clinical characteristics, one compared survival, and three compared both patient characteristics and survival. Differences were found between those patients with complete data and those with missing data in seven of these articles. In an additional article, the authors reported that there were no differences in characteristics between the complete cases and the whole sample.

## DISCUSSION

### Impact of missing data

Missing data can introduce bias depending on the missing data mechanism and the adopted missing data approach. This can lead to under- or overestimation of the parameter estimates, which may affect the prognostic ability of the covariates and change the study's conclusions. Excluding cases with missing data reduces the analysable sample size and wastes valuable information that has been collected. Consequently, the power to determine significant covariates is reduced, variances are overestimated and confidence intervals are too wide (Little and Rubin, 1987). Conversely, including all cases without accounting for the fact that the data were unknown, for example, with single imputation, overstates the available sample size, leading to an underestimation of the variance and hence too narrow confidence intervals and more significant *P*-values (Rubin, 1987). Multiple imputation results in valid statistical inferences that properly reflect uncertainty due to missing values (Schafer, 1997).

The amount of missing data apparent in a study can provide a measure of the quality of a study. Studies having a lot of missingness may be considered of poorer quality than those with none or limited missing data. It is the responsibility of the authors to provide sufficient information to allow readers the ability to make an assessment of the study's quality and hence reliability of the obtained results.

### Frequency and handling of missing covariate data

This review has established that missing covariate data is a common occurrence in cancer studies performing multivariate survival analyses. Missing data was an issue in almost all of the reviewed prognostic studies, but its impact was explored in only a minority. Only 10 out of the 100 articles explored the differences in characteristics or outcome between the cases with complete or incomplete data.

The most commonly reported methods for handling missing covariate data were complete case analysis and available case analysis, regardless of the amount of missingness or possible missing data mechanisms. It is likely that the majority of articles not reporting the methods for handling the missing covariate data used these simple deletion techniques as well.

Three articles applied informal single imputation approaches and then analysed the data, assuming that these values were the true observed values, without consideration of the problems associated with not accounting for the missing data uncertainty (Rubin, 1987). Only one article reported using a multiple imputation approach, which was used when there were five incomplete covariates with a range of missingness from 0.5 to 6%. No specific details of the multiple imputation approach were provided in the article, and the reference cited (Harrell and Shih, 2001) did not relate to multiple imputation. However, multiple imputation was performed in this study using the TRANSCAN function (Harrell, 2001) within S-Plus 2000 (Insightful Corp, Seattle, WA) (personal communication).

### Reporting missing covariate data

This review has highlighted important deficiencies in the reporting of missing covariate data. In the majority of articles the extent of missing covariate data is unclear. The methods used to handle missing covariate data were obtainable in only 40% of the articles with apparent missing data. Very few articles made it clear how many cases were actually analysed and the associated number of events. Only one article included a separate section devoted to missing data. We are concerned that very few authors have considered the impact of missing covariate data; it seems that missing data is generally either not recognised as an issue or considered a nuisance that it is best hidden.

It was not the objective of this study to consider the methodological issues regarding the construction of prognostic models or the appropriate results to report. Previous articles have provided recommendations for the reporting of studies using survival analyses (Altman *et al*, 1995) and specifically for the presenting of prognostic factor studies (Simon and Altman, 1994; Altman and Lyman, 1998; Riley *et al*, 2003). Unfortunately, the current review shows that earlier recommendations are still not generally followed, leaving the readers of many articles unclear about many important aspects regarding how the authors performed the survival analysis.

**Figure 2:**
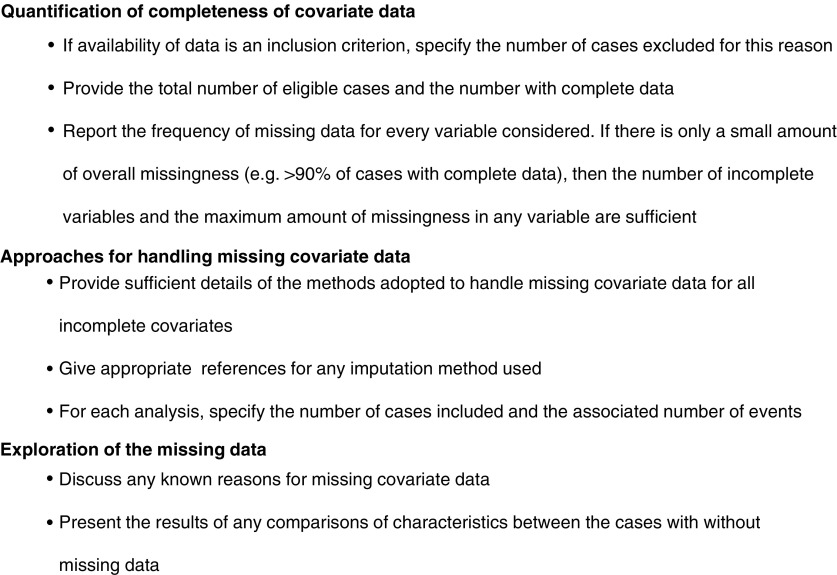
Guidelines for reporting prognostic studies with missing covariate data.

We are unaware of any guidelines specifically for the reporting of missing covariate data, although guidelines exist for reporting quality of life end points, which include that the amounts of missing data and their possible causes should be documented along with how they are analysed (Staquet *et al*, 1996). Therefore, we have proposed guidelines for reporting prognostic studies with missing covariate data (Figure 2

), which should supplement the specific recommendations for prognostic modelling (Simon and Altman, 1994; Altman and Lyman, 1998; Riley *et al*, 2003). For readers to quantify the completeness of covariate data, the number of cases deemed ineligible due to the unavailability of data should be provided. The total number of eligible cases and the number with complete data should also be stated, allowing the proportion of eligible cases with complete data to be calculated. Ideally, as previously stated in guidelines for reporting survival analyses (Altman *et al*, 1995), the frequency of missing data for every variable collected should be reported, which can easily be incorporated into a patient characteristics table. However, if there is only a small amount of overall missingness (e.g. >90% of cases with complete data), then the total number of incomplete variables and the maximum amount of missingness in any variable may suffice. The method adopted to handle missing data for every incomplete covariate should be clearly stated, with sufficient details of any imputation strategies to allow readers to know how the method was performed and, if desired, apply the methodology elsewhere. Appropriate references should be given for any imputation method used. For each analysis, the number of cases included and the associated number of events should also be provided, as previously suggested by Riley *et al* (2003). Finally, any known reasons for the missing data should be discussed and the results of any comparisons of characteristics between the cases with complete data and those with incomplete data presented. This information allows readers to make informed judgements regarding the potential missingness mechanism and hence the appropriateness of the applied methods.

**Figure 3:**
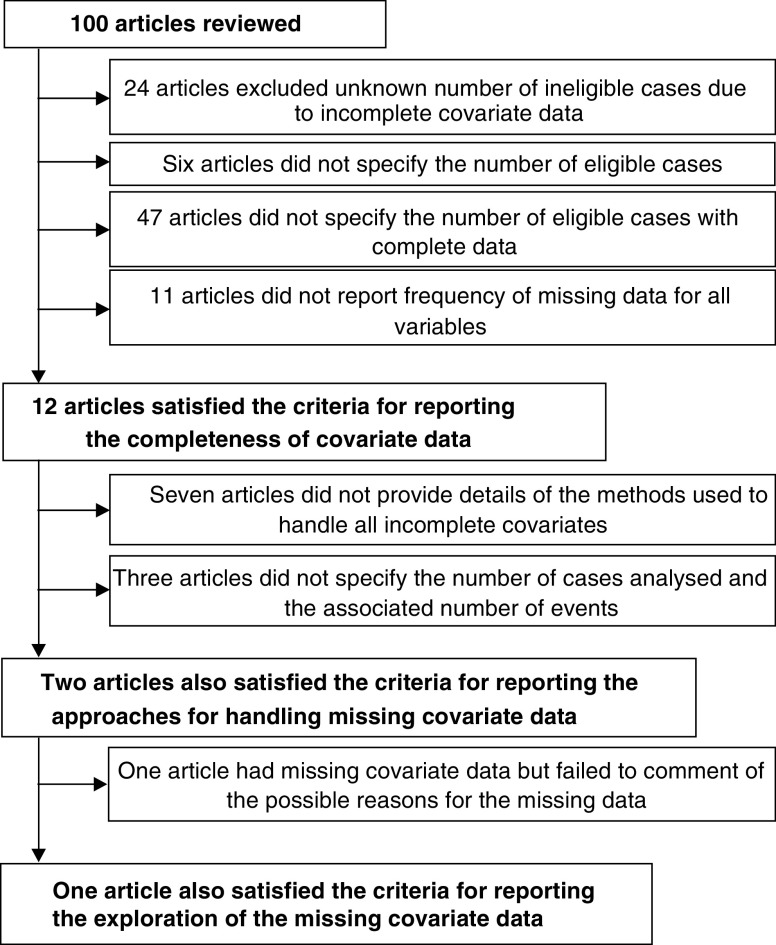
Flow diagram showing adherence to proposed guidelines.

The overall reporting quality of the 100 articles within the current review according to the suggested guidelines is displayed in Figure 3

. Only 12 studies would have satisfied the proposed guidelines for reporting the extent of missing covariate data and only two of these studies also complied with the recommendations for reporting the approaches used for handling incomplete covariate data. One of these studies had some missing covariate data, but unfortunately failed to comment on the possible mechanisms resulting in the missing data, and one was a randomised trial with no evidence of missing covariate data and therefore exploration of the missing data was not required. Therefore, only one article provided all the information considered important regarding the quantification, handling and exploration of missing covariate data, and hence satisfied the proposed guidelines for reporting missing covariate data when constructing prognostic models.

It is evident that better quality reporting of prognostic studies is required, especially with missing covariate data being an issue in the majority of reviewed studies. The proposed guidelines for presenting prognostic studies with missing covariate data, if followed, should help to contribute to the improved reporting in future articles.

## Appendix A

Summary reference details for the 100 articles reviewed are provided in Table A1

**Table A1:**

**Journal**	**Volume**	**Start page**
**Ann Oncol**	13	308,523,1017,1087,1275,1364,1454,1460,1550,1568,1621,1779
**Br J Cancer**	86	31,331,396,674,1899
**Br J Cancer**	87	8,281,756,772,1140,1404,1422
**Cancer**	94	14,125,264,344,352,386,421,658,746,752,854,873,940,973,1049,1121,1886,2180,2813,2836
**Eur J Cancer**	38	401,511,527,1059,1065,1181,1329,1343,1502,1860,1987
**Int J Cancer**	97	512,518,574,770
**Int J Cancer**	98	415,879,883,900,916
**Int J Cancer**	99	100,418,579,589,
**Int J Cancer**	100	290,452,463
**J Clin Oncol**	20	42,179,221,231,247,254,289,680,707,732,776,1353,1368,1721,1793,2076,2633,2672,2930,3972
**J Natl Cancer Inst**	94	26,116,284,490,662,1091,1160,1569,1635

.
